# Sustainable Development: Growing Green Communities

**Published:** 2005-05

**Authors:** Carol Potera

Advocates of green housing received a boost when the nonprofit Enterprise Foundation of Columbia, Maryland, announced that it plans to build 8,500 environmentally friendly, affordable homes through its Green Communities Initiative. Launched in September 2004, the Green Communities Initiative commits $550 million over five years to developers to construct housing units that promote health, conserve energy and natural resources, and are located near public transportation, jobs, social services, stores, and schools. The initiative is led by the Enterprise Foundation and the Natural Resources Defense Council, with the support of several other organizations.

The Denny Park Apartments, being built in Seattle, Washington, are a shining example of what can be achieved through the Green Communities Initiative. The project—the first recipient of funding through the Green Communities Initiative—is being built by the Low Income Housing Institute (LIHI), which develops and manages affordable housing units in Seattle. The six-story building will provide 50 units ranging from studios to three-bedroom apartments. The first tenants plan to move in by December 2005. Ten units will be reserved as transitional housing for homeless families.

The apartment building features numerous energy-saving features. It is located along an east–west axis to allow the units to capture more natural light through their oversized windows, reducing electricity bills. A central gas boiler will supply hot water and heat to all the units. “Gas is more efficient and less expensive than electricity in Seattle,” says architect Brian Sweeney, manager of development for LIHI. Moreover, hot-water heat makes people feel warmer at lower room temperatures than electric heat, according to Sweeney—people feel as warm at 65°F with hot-water heat as they do with drier electric heat set at 69–70°F. Ventilation fans will run continuously to reduce humidity and mold growth, a problem in Seattle’s moist climate.

The building is being constructed with sustainable building materials such as metal roofing and metal siding, which should last 50 years. These durable materials eliminate petroleum-based products such as traditional asphalt roofing shingles and oil-based exterior paint, which—in addition to their nonsustainable provenance—must be replaced every 10 years or so. The project is using caulks, paints, adhesives, and other construction materials with low levels of volatile organic compounds to ensure healthy indoor air. Carpets are made from recycled plastic products. Rainwater will be captured off the metal roof, purified by gravel filtration, and recycled to irrigate the landscaping, including a communal garden for the tenants.

Although green buildings currently can cost about 2% more to construct, the self-evident long-term energy and health benefits are passed on to tenants. “The things considered ‘green’ today are going to be part of any building project in the next ten to fifteen years,” predicts Sweeney.

Dana Bourland, senior program director at the Enterprise Foundation, says the foundation has received about 50 letters of inquiry from public housing administrators across the United States. Eight grants have been awarded for other projects in the Bronx, Boston, Chicago, and other cities, which are in various stages of development. The housing projects can consist of multi-family or single-family structures, but individuals cannot apply to build just one home. Most of the applicants are public housing offices and nonprofit groups seeking to improve their communities.

What distinguishes the Green Communities Initiative from other green housing programs? “We’re not interested in just one aspect like energy efficiency,” says Bourland. Each project has to meet “certain levels of greenness,” she says. Her group’s criteria include meeting standards for water conservation, healthy indoor air, use of environmentally friendly materials, good operations and management (for example, making sure gutters that collect rainwater for irrigation are kept free of leaves), and optimal location (for example, projects located within a quarter-mile of public transportation earn extra points toward meeting funding criteria). “Our goal is to transform the marketplace and shift the way we build to achieve health, environmental, and economic benefits in communities,” says Bourland.

## Figures and Tables

**Figure:**
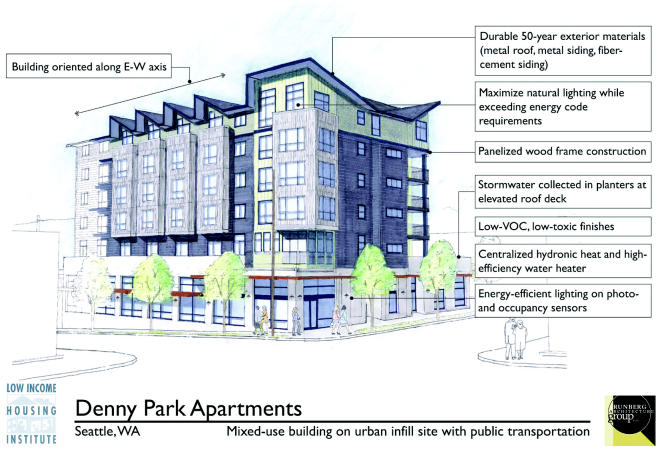
Sustainable Development Features

